# Investigations on Vector-Borne and Aerosol Transmission Potential of Kaeng Khoi Virus in Cave-Dwelling Wrinkle-Lipped Free-Tailed Bats (*Chaerephon plicatus*) in Thailand

**DOI:** 10.3390/microorganisms9102022

**Published:** 2021-09-24

**Authors:** William A. Neill, Rebekah C. Kading

**Affiliations:** 1 Department of Molecular Microbiology and Immunology, Johns Hopkins School of Public Health, Baltimore, MD 21205, USA; neillwa43@gmail.com; 2 Department of Microbiology, Immunology, and Pathology, College of Veterinary Medicine and Biological Sciences, Colorado State University, Fort Collins, CO 80523, USA

**Keywords:** emerging arbovirus, chiroptera, ectoparasite, bat bugs, vector competence, bunyavirus

## Abstract

Kaeng Khoi virus (KKV; Order: *Bunyavirales*, Family: *Peribunyaviridae*, Genus: *Orthobunyavirus*), is an endemic viral infection of the wrinkle-lipped free-tailed bat (*Chaerephon plicatus*; also known as *Tadarida plicata plicata*). Viral isolates from bat bugs (Family: *Cimicidae*) suggest vector-borne transmission, but in general little is known about the ecology of KKV and seroprevalence in the local human and animal populations. Transmission studies and a serosurvey were carried out in Kaeng Khoi cave, Saraburi province, Thailand, during 1973–1974. Experimental transmission studies were performed with bat bugs captured within the cave to determine the potential for vector-borne transmission, and sentinel laboratory mice placed inside arthropod-proof cages within the cave to assess the potential for aerosolized transmission. Antibodies to KKV were detected in roof rats (*Rattus rattus*) inhabiting the cave, in dogs living in the valley, and in humans. Freshly collected cimicids were positive for KKV, but the virus did not replicate in laboratory-inoculated bugs. Sentinel mice placed in Kaeng Khoi cave in open cages consistently became infected with KKV, as determined by the development of neutralizing antibodies. Mice placed in arthropod-proof cages also developed antibodies, indicating the possibility of airborne transmission of KKV.

## 1. Introduction

In 1969, a virus new to science, affecting wrinkle-lipped free-tailed bats (*Chaerephon plicatus*; also known as *Tadarida plicata plicata*), was discovered in Sara Buri province, Thailand. The virus was identified morphologically as a member of the now-recognized order *Bunyavirales* and within this group it was a serologically unique virus (Appendix A). The new virus was named Kaeng Khoi virus (KKV) after the locality of first isolation [1]. Kaeng Khoi virus was repeatedly isolated from dead free-tailed bats and arthropods collected in Kaeng Khoi cave [1,2] and has since also been isolated from bats (*Chaerephon plicata*) in Cambodia [3] and bat flies (*Eucampsipoda sundaica*) associated with bats in the genus *Rousettus* in the Yunnan Province of China [4]. Neutralizing antibodies to KKV were detected in the sera of people who mined guano from Kaeng Khoi cave [1] demonstrating that people are exposed to this virus, although the pathogenic potential remains unknown.

The bunyaviruses comprise a large and taxonomically diverse group of viruses with over 450 unique viral species from 12 recognized families [5,6]. Transmission of bunyaviruses is typically through an arthropod vector, with the exception of hantaviruses and arenaviruses which are transmitted by aerosolization of viral particles in the feces and urine of rodents. Many orthobunyaviruses in the family *Peribunyaviridae* are vectored by mosquitoes or midges; nairoviruses by ticks; and phenuiviruses by sand flies, midges, ticks or mosquitoes [7]. Bunyaviruses in the family *Tospoviridae* are recognized plant-pathogens vectored by thrips [7]. Hemorrhagic fever viruses such as Crimean Congo hemorrhagic fever virus (CCHFV) and Rift Valley fever phlebovirus (RVFV) (Family: *Phenuiviridae*) can also spread by contact with contaminated fluids [8,9]. Rift Valley fever phlebovirus has an altered pathogenesis when transmitted by aerosol [10].

Within the family *Peribunyaviridae*, KKV clusters phylogenetically with Mojuí dos Campos virus (MDCV) which is associated with bats in Brazil [11] and Nyando virus, which has been isolated from mosquitoes throughout Africa and is known to cause febrile disease in humans [12,13,14,15]. The phylogeographic disparity among viruses in this clade, as well as the connection of MDCV and KKV with bats, raise questions about the ecology and potential vector-borne transmission of these bat-associated viruses. The study of viral transmission among bats by arthropod ectoparasites is a burgeoning field and has recently been reviewed by Fagre and Kading [16]. Wingless bat flies in the family *Nycteribiidae* have been found infected with a diversity of ledanteviruses (Family: *Rhabdoviridae*) [17,18], apicomplexan parasites in the genus *Polychromophilus* [19,20], and bacterial species in the genus *Bartonella* [21,22,23]. Schuh et al. [24] recently demonstrated the role of soft ticks in circulating Kasokero virus (Order: *Bunyavirales*, Family: *Nairoviridae*) among Egyptian rousette bats (*Rousettus aegyptiacus*) in Uganda. The association of Kasokero virus with a tick vector was not surprising, given the established association of Crimean Congo hemorrhagic fever virus (Order: *Bunyavirales*, Family: *Nairoviridae*) with ticks [25]. In contrast, bugs in the family *Cimicidae* (Order: *Hemiptera*), while hematophagous, have been implicated in the transmission of very few infectious agents. Human bed bugs (*Cimex lectularius*) are not known to be responsible for the biological transmission of any pathogens [26], although they can be infected with some microbes, including *Borrelia recurrentis* [27] and *Bartonella quintana* [28] bacteria in the laboratory, and are capable of mechanically transmitting hepatitis B virus [29,30]. The alphaviruses (Family: *Togaviridae*) Fort Morgan virus and Buggy Creek virus, reservoired in birds, are examples of viruses maintained in a vertebrate host and transmitted by a cimicid vector [31,32]. Cimicids have been long known to parasitize bats, but their role as a primary vector of a bat-associated infectious agent is yet to be characterized.

Given the presence of diverse ectoparasites in Kaeng Khoi cave and the isolation of KKV from bats, the aim of the present study was to understand how the KKV is maintained in the free-tailed bat population, including a possible role for vector-borne as well as aerosol or contact modes of transmission. This study had three aims: (1) Determine seroprevalence of KKV in humans and cave-dwelling animals, (2) Perform viral transmission studies with field-collected cimicids, and (3) Evaluate potential for aerosol transmission through the placement of sentinel mice in Kaeng Khoi cave.

## 2. Materials and Methods

A description of the original characterization of KKV, and virus isolation procedures used in these studies are provided in Appendix A.

### 2.1. Description of the Study Area

The Kaeng Khoi cave (14°35′ N; 101°8′ E) [1] is situated in a limestone mountain range which marks the division between the fertile Chao Phraya Delta region of Central Thailand and the northeastern Korat Plateau. Six mountains in the region, ranging in height from 563 to 616 m above sea leve1, form a fertile, landlocked valley 100 m above sea level. These cavernous foothills are characterized by steep precipices and limestone crags in the upper portions. The vegetation in these upper portions includes a variety of trees and succulent shrubs (*Dracaena* sp.), cycads (*Cycas pectinata)*, and antique spurge (*Euphorbia antiquorum*). The vegetation of the lower slopes consists of mixed deciduous trees and grasses. The climate is tropical and wet with a distinct dry season. The mean annual temperature for the year 1967 was 27 °C. There are two seasons; a dry season usually of about 5 months in duration (October–February) and a wet season of about 7 months in duration (March–September). Annual rainfall for 1969 was 168 cm with about 94% of the rain falling during the wet season.

**Figure 1:**
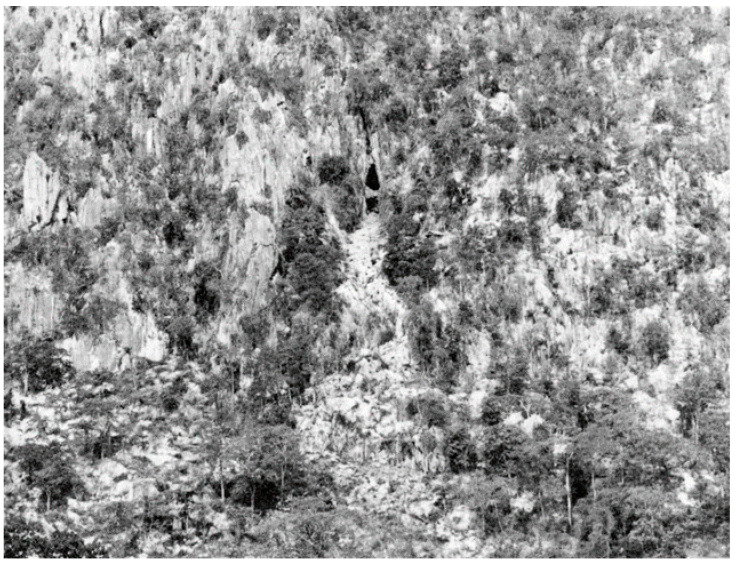
Kaeng Khoi cave, 1969–1970.

The Kaeng Khoi cave is located 180 m above the valley floor on the steep western face of Khao Lorn Phat, a mountain which forms part of the eastern border of the valley (Figure 1). The cave has two exits; the main one faces west, and the other is a hole in the ceiling which exits from the rear of the large central room in the cave. The cave is divided into six rooms by limestone abutments. Five rooms on the periphery are connected to the large central room at different levels by means of large, walkable passageways. Rooms 1 and 2 were the areas utilized in this study. These rooms were inhabited all year by large bat roosts and functioned as maternity rooms. Temperatures in these rooms, as measured by 7-day hygrothermographs during January–April 1970, remained essentially constant (26.9 ± l.4 °C). In the same period, there was maximal variation in the valley temperatures. The relative humidity in the cave varied from 50 to 94% during January–April 1970.

The species of mammals, their identified ectoparasites, and birds that preyed on exiting free-tailed bats are listed in Table S1. The wrinkle-lipped free-tailed bat (*Chaerephon plicatus*, also known as *Tadarida plicata plicata*) was the most numerous of the vertebrates with a population approaching one million. The population of tomb bats (*Taphozous theobaldi*) was much lower and was estimated to be <100,000.

Approximately 200 people live in bamboo thatched houses clustered along the valley’s two central roads and in the surrounding mountain valleys. About fifty people farm plots of land which produce corn and banana crops. Some of the people work in the Kaeng Khoi cave, sweeping up the freshly fallen bat guano. The guano is stored in two separate roofed bamboo huts in the valley. The valley residents keep few domestic animals, mostly dogs, pigs and chickens.

**Figure 2:**
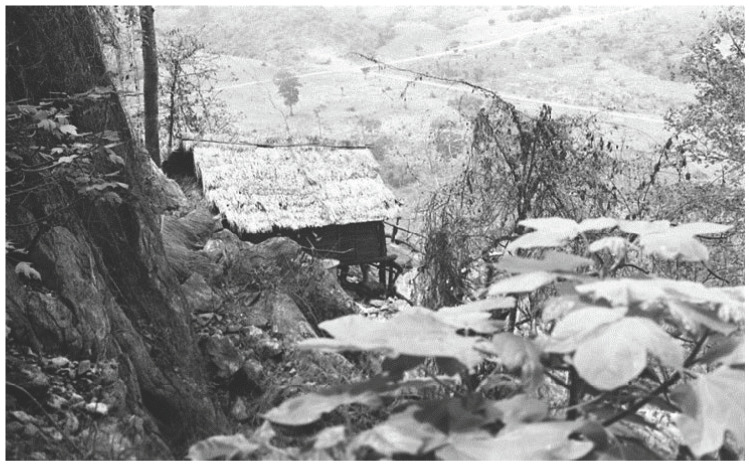
House and workspace constructed in 1973 outside the cave, to facilitate the onsite processing of samples.

To facilitate field work, a small bamboo research hut was built near the main entrance of the cave (Figure 2). Furnishings included large water vessels, an electric generator, lights, a centrifuge, a cot, and the equipment necessary to process the collected specimens.

### 2.2. Seroprevalence of KKV in Humans and Cave-Dwelling Animals

Normal adult and newborn bats were caught by hand in the mid-morning hours (0600–0800) from rooms 1 and 2. They were then bled, sexed and individually identified. Morbid and dead bats were collected from the floors of rooms 1, 2, and 3.

To collect live rodents, traps constructed from 13 × 13 × 41 cm heavy gauge wire mesh were baited with bananas and placed in rooms 1, 2, and 3 of the cave or outside the cave within a 30 m radius of the entrance. Trapped rodents were bled, sexed and estimated for age under light ether anesthesia. Mammals and birds were identified by Dr. Joe T. Marshall of the Mammalogy-Ecology Section of the South-East Asia Treaty Organization (SEATO), renamed in 1977 as the Armed Forces Research Institute of Medical Science (AFRIMS).

Blood from the rodents and adult bats was drawn by cardiac puncture with 2.5 mL disposable syringes. The sera from bats were diluted (1:2) at the time of bleeding by drawing the blood into syringes containing appropriate volumes (0.5 mL for adult and 0.25 mL for newborn) of sterile tissue culture growth medium 199 (GM). Dogs were bled from the cephalic vein. Oral swabs from bats were collected by massaging the oral cavity with a cotton swab. Urine was collected from handheld urinating bats with Pasteur pipettes. Oral swabs and urine samples were diluted in l.0 mL GM. All specimens were stored on dry ice at the field station until their transfer to the main laboratory at SEATO (approximately a 2-h drive), where the sera were stored at −20 °C and the blood clots, oral swabs, urines and animal bodies at −60 °C.

Guano miners working in five separate caves scattered within a 120 km radius of Bangkok were bled for serologic studies. In addition, sera were collected from residents of Kaeng Khoi valley who did not give a history of working in Kaeng Khoi cave. Age, sex and history of work in the caves were recorded for each serum donor.

#### Serologic Tests

Neutralization tests for antibodies against KKV were performed by plaque reduction or in tube cultures using Vero cell monolayers. Sera were inactivated at 56 °C for 30 min and diluted with GM. Virus was diluted to give an effective virus dose of 100 TCID_50_ (50% tissue culture infectious dose) or 50–180 p.f.u. (plaque-forming units) (Appendix A). A 0.4 mL volume of each serum dilution was mixed with an equal volume of an appropriate virus dilution. The serum-virus mixture was incubated for 1 h at 37 °C. Following incubation, two drained 1 oz. prescription bottles were inoculated, each with 0.3 mL of serum-virus mixture. After adsorption at 37 °C for 1 h, 8–10 mL of overlay medium was added to each flask. The overlay medium consisted of equal volumes of sterile 2% Ionagar No.2 (Oxoid Division, Oxo Ltd., London, UK) [33] in distilled water and fluid medium containing the following presterilized components: 51.3 mL heat-inactivated (at 56 °C for 30 min) fetal calf serum; 1.2 mL of a 5% yeast extract; 3 mL of a 10% lactalbumin hydrolysate; 5.3 mL NaHCO_3_ solution (7.5% stock); 1.8 mL l00× penicillin-G and streptomycin; and 3 mL neutral red (1:1000). Drained tubes were inoculated with 0.1 mL of serum–virus mixture.

After absorption at 37 °C for 1 h, 1 mL of maintenance medium was added to each tube. Roux culture bottles and tubes were incubated at 37 °C and observed, respectively, for plaques and CPE on day five. A plaque reduction of 80% or more and no CPE in tubes were the criterion for a positive test.

Serologic examination of human sera for antibodies to Japanese encephalitis virus (JEV) and chikungunya viruses (CHIKV) was performed by the microtiter HAI test [34] Both JEV and CHIKV are endemic to this region; we sought to compare the seroprevalence of these two arboviruses relative to that of KKV. Our hypothesis was that seroprevalence for KKV would be higher in guano collectors due to higher exposure rates from cave-entering frequency as opposed to seroprevalence for JEV and CHIKV that would be similar across all ages and activities. These tests were performed by the personnel of the SEATO Department of Virology.

### 2.3. Experimental Infections of Bat Bugs

Attempts were made to infect cimicids by feeding them on viremic mice. The cimicids were concentrated on because they were abundant and they feed on both species of bats, while ticks, bat flies, and fleas were found associated with only one or the other bat species (Table S1). Neutralizing antibodies against KKV were found in both species of bat. A single pool of the prototype Kaeng Khoi strain, S-19-B, originally isolated from the brain of a dead free-tailed bat in 1969 (Appendix A), was used for all mouse inoculations. The inoculum used in these studies was prepared from the supernatant of Vero cells infected with the ninth suckling mouse brain (SMB) passage of KKV strain S-19-B.

#### 2.3.1. Attempts to Demonstrate Virus in Cave Cimicids

The cimicids were *Cimex insuetus* Ueshima and *Stricticimex parvus* Ueshima, with both of these new species being identified from the Kaeng Khoi cave [35]. The adult cimicids (not identified by species) from the cave were placed in half pint wide mouth glass containers with screened tops and maintained in an incubator at a temperature of 25 °C. These cimicids were either used on the day of collection or after five days of starvation. A collection of 900 adult cimicids was divided into two groups, one for virus isolation attempts and the other for attempts to transmit infection to suckling mice. The first group of 400 cimicids was subdivided into 20 pools and each pool was individually triturated in sterile 7 mL Ten Broeck tissue grinders. The resultant suspension was centrifuged at 9750× *g* for 30 min at 4 °C. The supernatant was withdrawn and divided into two parts, one portion was frozen at −60 °C for reisolation attempts, and the other was inoculated into suckling mice, i.c., for virus isolation. The second group, totaling 500 cimicids, was first starved for five days, and then divided equally into 25 pools. Each pool of 20 starved cimicids was allowed to feed on a normal suckling mouse. The cimicids fed readily. The mice identified by the pool of feeding cimicids, were observed twice daily for 21 days for mortality.

#### 2.3.2. Attempts to Infect Cimicids

Suckling mice were inoculated with 200 SMLD_50_ of KKV strain S-19-B and were collected when moribund. About 35 cimicids collected from Kaeng Khoi cave and starved for five days were allowed to feed for 45 min on these mice. Cimicids which had fed were maintained in an incubator at 25 °C and re-fed on normal suckling mice every 7 days. Three cimicids were collected at each of the following time intervals after feeding: one hour, and on days l, 3, 15, 16, 18, 20, 22 and 29. Each pool of three cimicids was triturated in 1 mL of GM in 7 mL Ten Broeck tissue grinders. Undiluted and 10-fold dilutions of the supernatant were tested for virus by i.c. inoculation of suckling mice. The mice were held for 15 days and examined daily for mortality.

### 2.4. Assessment of Aerosol Transmission Potential

To determine whether or not aerosol transmission inside the cave was possible, sentinel mice were placed in open or arthropod-proof cages for viral infection. Weanling Swiss mice derived from the SEATO mouse colony were placed in arthropod-proof cages (APC) or open cages (OC). The latter type of cage allowed free entry of arthropods (Figure S1). The APC were constructed from polyvinyl chloride (PVC) 10.6 cm standard tubing and T-junctions (Figure S2). Arthropod barriers were made of a combination of cotton cloth (92 mesh, 0.15 × 0.19 mm openings) and fine mosquito screening (15 mesh). Coarse heavy-duty screens (21 mesh) were installed to protect the barriers. The barriers were sealed onto both ends of the animal chamber by sleeves and PVC sealer. Two chambers, one for food and the other to house the mice, were inserted into the T-junctions and sealed with surgical tape. Water was delivered by two straight 3-inch stainless steel sipper tubes. A filter consisting of a 92 mesh cloth was sealed to the 6 mm rubber stopper that connected each of the removable 8 oz. glass water bottles to the sippers. A small battery-operated fan was inserted into one end of the animal chamber for air circulation. The open cages were cylindrical (Greiner Sci. Corp.), 21.6 cm in diameter and 23 cm high. The sides and floor were constructed from 3 mesh 18 gauge wire, the top being covered with a metal lid. Food was placed loose inside the cage and water was supplied by an 8 oz glass water bottle. The APC cages held 10 Swiss mice and the OC cages held 6 female Swiss mice (3–4 weeks old) per cage. Mice were exposed on the floors or walls of rooms 1 and 2 for varying periods of time. After exposure, the mice were brought back to the main laboratory and held for 15 days, after which they were exsanguinated. The sera were tested individually for the presence and titers of neutralizing antibody against KKV.

## 3. Results

### 3.1. Antibody Prevalence in Other Vertebrates

**Figure 3:**
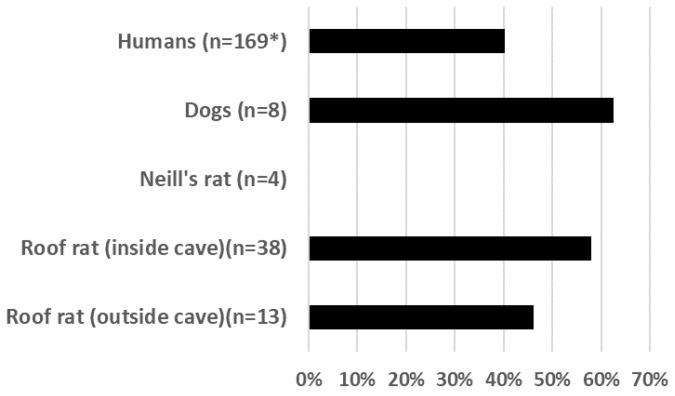
Summary of antibody prevalence for KKV among humans, rats, and dogs around Kaeng Khoi cave. * Human seroprevalence reported here represents an average of the data presented in Table 1.

Antibody prevalence against KKV was high across diverse vertebrate taxa associated with Kaeng Khoi cave (Figure 3).

#### 3.1.1. Rodents

One species of rodent, the roof rat (*Rattus rattus*) was trapped in the cave. These rats were active in all major rooms, though it was most noticeable in the main central room. It was regularly noted scavenging on morbid bats. In February 1974, 45 traps were set daily for five consecutive days; a total of 38 roof rats were trapped. Rodents of two additional species were trapped outside the cave: the limestone rat (*Niviventer hinpoon*) and Neill’s long-tailed giant rat (*Leopoldamys neilli*).

Tests for neutralizing antibodies against KKV in rodents were done at a single 1:10 dilution of the sera. The antibody prevalence was related to the age of the roof rat; it was 82% (18/22) in adult and 27% (4/16) in the juvenile rats. Six of the 13 roof rats and none of the four tested Neill’s long-tailed giant rats trapped outside the cave had antibodies. Sera from the limestone rat were not tested.

#### 3.1.2. Dogs

Five of eight dogs captured within a half kilometer radius of the two guano storage huts had serum neutralizing antibodies to KKV at a dilution of 1:10. Dogs were not able to climb the steep rock face leading to the cave, but were observed scavenging on dead bats in the guano huts.

#### 3.1.3. Humans

Two groups of sera were collected, one of 108 from guano miners from five separate caves (including Kaeng Khoi cave), and the other of 61 from residents of Kaeng Khoi valley who did not give a history of working in the cave. Neutralizing antibody prevalence in the workers of the five guano-mining groups varied from 43% to 78% (Table 1), with a combined prevalence for all guano miners of 57%. The Kaeng Khoi valley residents without mining histories had an antibody prevalence of 10% compared with 61% in Kaeng Khoi valley residents who were guano miners. In each of the five localities, the guano miners gave a history of working only in the cave in their own area. The data, therefore, indicate that infection with KKV is widespread in free-tailed bats in Central Thailand. The antibody prevalence was clearly related to the length of exposure to working in caves (Figure S3). Antibodies were found in 100% of those miners who worked for seven or more years. The most reliable histories were available for the guano miners in Kaeng Khoi valley. In this group, antibodies to KKV were detected in all of 23 individuals who worked in the cave for one or more years and in four of 21 who worked for less than one year.

**Figure 4:**
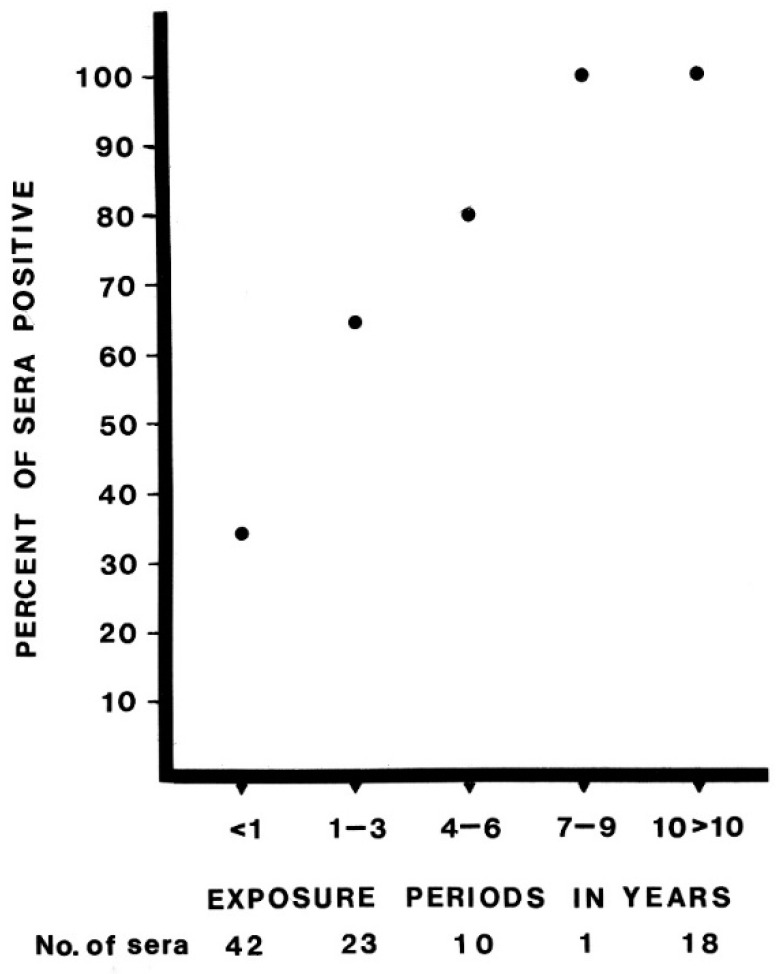
Neutralizing antibody prevalence against KKV in guano miners from five caves by number of years the individuals reported to work as guano miners.

The guano miners as a group were older than residents from Kaeng Khoi valley. Therefore, the antibody prevalence to KKV in the two groups was examined by age. Further, the sera from both groups were tested for the presence of HI antibodies to chikungunya (CHIKV) and Japanese encephalitis (JE) viruses, both of which are arboviruses endemic to the area. The results are given in Table 2. The prevalence of antibodies against CHIKV and JE were essentially similar in the two groups, whereas antibodies against KKV were more prevalent in the guano miners in each age group. While the antibody prevalence for the two groups CHIKV and JE were similar for the two groups, antibodies against KKV were much more frequent in the guano miners as compared to residents (Table 2) and antibody prevalence in guano miners increased with age (Figure 4).

### 3.2. Viral Transmission Studies

#### 3.2.1. Transmission Studies with Cimicids

**Figure 5:**
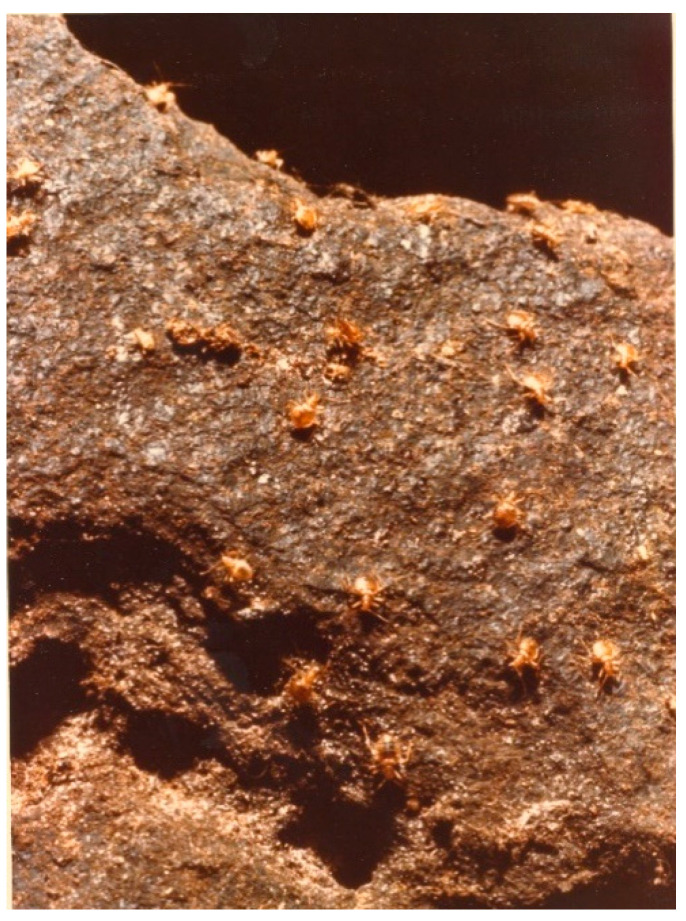
Cimicid bugs on the cave wall, Kaeng Khoi cave. A flea perched on top of a bug is also present mid-way down the center of this photo. Photo by W.A. Neill.

Attempts were made to test for presence of virus in the cimicids, either by direct inoculation of cimicid suspensions into mice or by permitting the cimicids to feed on suckling mice. Bugs present on the cave wall are pictured in Figure 5. A total of 900 adult cimicids, engorged or non-engorged, were collected in March 1973. Four hundred of these were divided into 20 pools, each of 20 cimicids, for mouse inoculation. The remaining 500 cimicids were starved for five days and fed on suckling mice. Each group of 20 cimicids was allowed to feed on three mice in a cage until all individuals of the group were engorged. This took about two hours. The 75 mice fed on by the cimicids were observed for 21 days for virus-specific mortality.

Of the 20 pools inoculated into mice, seven yielded KKV. No mortality was observed in the 75 mice fed on by the cimicids.

The presence of virus in the freshly captured adult cimicids could have been a result of their having fed recently on viremic bats. To determine if virus multiplication occurred in the cimicids, starved adult cimicids were fed on viremic mice and tested at 1 h, 1 day, 3 days, 15, 16, 18, 22, and 29 days for the presence of virus, over a period of 29 days (Table 3). A group of three cimicids was tested each time. The cimicids ingested a large amount of virus, as shown by the titration at one hour post feeding (Table 3). The virus dropped by about 1.5 log_10_ SM LD_50_ by 24 h, and was not detectable in the tests on day 3 and thereafter.

These data do not suggest a biological role for cimicids in transmission of KKV.

#### 3.2.2. Transmission Studies with Sentinel Mice

The sentinel mice in either OC or APC were exposed to the cave environment at various times of year. The surviving mice were observed in the laboratory after 15 days and then bled for antibody determinations of individual sera.

The results are summarized in Table 4 in terms of proportions of exposed mice for which antibodies to KKV were present after cave exposure, as well as of the proportion of cages in which at least one mouse developed antibodies. In room 1, in OC, 50% of mice were infected in 2.8 days in June 1973, and 100% in 19 days in March 1973. In January–February of 1974, only 27% of the mice were infected after an 18-day exposure in OC. A single observation was made in room 2 in June 1973. Twenty-nine percent of the mice had antibodies, lower than that of 56% obtained for mice exposed in room 1 in the same time period. At least one mouse became infected in each of the 19 open cages which were exposed during these time periods.

These data show that viral transmission likely occurred in the cave during short exposure periods in June (2-to-8 days) and that the observed transmission rates were lowest in January-February and highest during March through June. Viral transmission was not studied at other times of the year.

A single experiment was performed with APC. A total of 23 mice in three cages were exposed in room 1 for 19 days in March 1973. A total of eight mice (35%) developed antibodies; at least one mouse in each of the three cages was antibody-positive. As described above, the conversion rate for the same exposure in OC was 100%.

## 4. Discussion

Several important components of the epidemiology and transmission of KKV infection of the Thailand free-tailed bat inhabiting Kaeng Khoi cave were studied during 1973–1974. Here we report on possible vector-borne and aerosol modes of viral transmission and dissemination of KKV from the free-tailed bat communities to other mammals, including humans. The Kaeng Khoi cave has large populations of actively feeding ticks, fleas, and cimicids all year round, so that arthropod-borne transmission of KKV could occur if any of the above arthropods were a biological vector. Ticks as well as the fleas and the bat flies were apparently host-specific (Table S1), while both species of bat have antibodies to KKV. The cimicids, because they feed on bats of both species, were hypothesized to be the best candidates for possible arthropod transmission of KKV. Bat flies in the family *Nycteribiidae* have since been found infected with KKV in the Yunnan Province of China [4]. Additional newly-recognized orthobunyaviruses closely related to KKV have also been isolated from bat flies in the genus *Eucampsipoda* in both China [36] and South Africa [37]. The virus from China is believed to be a divergent strain of KKV [36] while the virus from South Africa, named Wolkberg virus (WBV), is unique but also clusters in the Nyando serogroup with KKV and MDCV [37]. Still, the isolation of virus from these insects alone does not implicate them as vectors. The results of experimental infection with the cimicids in this study demonstrated that while the bugs may imbibe KKV while feeding on viremic bats, KKV does not replicate in these arthropods, indicating that they are unlikely to be the biological vectors of KKV. It is still possible that they could infect susceptible bats mechanically through interrupted feeding, as infectious virus was detected in experimentally exposed bugs up to one day post-exposure (Table 3). These results are akin to those obtained by Jupp and McElligott [30] who demonstrated the persistence of surface antigen of hepatitis B virus in bed bugs (*Cimex lectularius*) for over 7 weeks, but found a lack of viral replication and biological transmission by the bugs. Mechanical transmission was possible, however, by bugs who excreted viral particles in their feces while feeding or who were interrupted in feeding and attempted a subsequent blood meal [29]. Viral particles were not found in the salivary glands of bed bugs [29]. Mechanical transmission could similarly explain KKV transmission among bats by cimicids. Alternatively, the natural vector may be a different arthropod such as bat flies, although bat flies were not found on the wrinkle-lipped bats in Kaeng Khoi cave (Table S1).

Contrary to the typical and expected arthropod-borne transmission of viruses in the genus *Orthobunyavirus*, it appears that KKV transmission in the cave could also occur by the respiratory route. KKV isolations from the urine and oral swabs of moribund bats demonstrate that virus is shed from an infected bat in urine and throat secretions ([1], Appendix A). We hypothesize that the high frequency vocalizations of the free-tailed bat could aerosolize their throat secretions, thereby making airborne transmission of KKV possible. Vocal communication between mother and infant bats has been well documented [38,39]. Human vocalizations are also known to be associated with the spread of respiratory pathogens [40,41]. Yan et al. [40] detected the RNA of influenza viruses in the exhaled breath of symptomatic human patients, and that coughing and sneezing was not essential for viral transmission. Airborne droplets generated during normal speech can also persist for tens of minutes in confined spaces, which would facilitate the transmission of infectious agents [42]. Contact transmission by large droplets of virus containing saliva or urine, without aerosolization, could also readily occur in view of the high density of bats (300/sq. ft) in the roost, although this should be further investigated.

Seroconversion of sentinel mice exposed in the APC provides further support for potential transmission of KKV by a non-vector-borne route. One limitation of these data are that true seroconversion cannot be claimed without serology data on the weanling (3–4 week old) mice prior to placement in the cave. Still, mice in each cage tested positive for neutralizing antibodies in the absence of arthropod exposure. Additionally, if infectious particles from bat urine or other secretions were still able to enter the APC, exposure may have happened through droplet contact. Mice in the APC were, however, not as readily infected as mice in the open cages. This may be due to one, or a combination of the following: (i) more air is circulated through the open cages; (ii) the barriers of the APC filter out large infectious droplets that are not truly airborne; (iii) cave arthropods also have a role in the transmission of KKV in the open cages. Additional studies on the potential for airborne transmission of KKV are warranted. Collectively, these data suggest that transmission of KKV is occurring by a non-vector-borne route. Transmission of KKV probably occurs throughout the year. Infection of sentinel mice, attempted at different times between January and June, was always successful.

The presence of neutralizing antibodies to KKV in the roof rat and the guano miners, together with the increasing prevalence of immunity, approaching 100% with longer exposure periods (Figure 4), show that KKV can be readily transmitted to these mammals and probably others that frequent these bat caves. The pathogenicity of infection in humans and roof rats is not known. The possibility remains that cross-reactivity of antibodies to other orthobunyaviruses endemic to the area may confound these results. At the time of virus characterization (Appendix A), no serological cross-reactivity was detected when the 1:4 dilution of hyperimmune mouse ascitic fluid to KKV was tested by complement fixation against 1:4 dilutions of antigens of arenaviruses, bunyaviruses, herpesviruses, orbiviruses, paramyxoviruses, picornaviruses, poxviruses, reoviruses, rhabdoviruses, togaviruses, nor to 67 ungrouped viruses and six minor grouped viruses, available at Yale Arbovirus Research Unit (Tables S2 and S3).

One mode of dissemination that should be further investigated is through the scavenging habits of animals. The presence of neutralizing antibodies to KKV in five of eight dogs, bled within a half kilometer radius of the two guano storage huts in the Kaeng Khoi valley, is possibly a result of the dogs scavenging dead bats in the guano huts, but this is still speculative.

In conclusion, this study challenges the assumption that KKV, like typical orthobunyaviruses, is strictly vector-borne. While arthropods may play some role in the mechanical transmission of this virus, the gregarious nature of these free-tailed bats may facilitate viral transmission by contact and airborne routes resulting in high seroprevalence among bats as well as spillover to humans and other animals entering the cave. The health significance of this cross-species transmission is not yet known, but should be investigated further given the diverse taxonomic range of vertebrate hosts KKV is capable of infecting as well as evidence that this virus is also pathogenic to the bats themselves (Neill and Kading, submitted). Even so, the ecological importance and economic value of these bats is not to be underestimated, as their guano serves as a significant source of plant fertilizer to the local community in addition to their consumption of agricultural pests [43,44]. Annual guano harvests in caves inhabited by wrinkle-lipped free-tailed bats can range from 40–70 tons per year [44]. Respiratory protection for guano miners and other people entering caves such as Kaeng Khoi is encouraged to live safely with bats, prevent cross-species transmission, and preserve this valuable resource.

## 5. Conclusions

1. Neutralizing antibodies to KKV were detected in roof rats inhabiting the cave, dogs in the valley and in humans. The prevalence of antibodies in humans was related to the length of exposure to the cave.
2. Virus was isolated from pools of recently collected cimicids, but experimental infection of cimicids did not reveal virus multiplication in these arthropods.
3. Sentinel mice in arthropod-proof cages tested positive for neutralizing antibodies after exposure to the cave environment.

## Figures and Tables

**Table 1:** Distribution of neutralizing antibodies against KKV in guano miners (GM) from five caves and in Kaeng Khoi valley residents.

			Number of Positive Sera					
			Antibody Titers					
Area	Sample Total	Number (%) Positive	10	10 *	20	40	80	160
Tham Thava (GM)	18	12 (67)	3	1	1	3	2	2
Wat Kao Chong Phram (GM)	23	10 (43)	6	2	1	1	-	-
Tham Wat Kao Wong Kat (GM)	9	7 (78)	2	-	3	-	2	-
Tham Kao Kad (GM)	14	6 (43)	6	-	-	-	-	-
Tham Kaeng Khoi (GM)	44	26 (61)	7	11	2	1	3	2
Kaeng Khoi valley residents (Non-GM)	61	6 (10)	-	6	-	-	-	-

* Screened at a 1:10 dilution only.

**Table 2:** Antibody prevalence to KKV, CHIKV, and JEV in guano miners and valley residents, by age. N = neutralization; HAI = hemagglutination inhibition.

	% Guano Miners with Antibodies				% Valley Residents with Antibodies			
Age Groups	No. in Age Group	KKV (N)	CHIKV (HAI)	JEV (HAI)	No.in Age Group	KKV (N)	CHIKV (HAI)	JEV (HAI)
5–16	33	30	24	46	35	3	11	20
17–40	49	63	51	80	13	31	69	77
40+	26	81	85	81	6	16	100	67

**Table 3:** Amount of virus in pools of three cimicid bugs at different times after an infectious blood meal with KKV.

Time Post-Feeding	Amount of Virus SM (i.c.) LD_50_
1 h	10 ^6.8^
1 day	10 ^5.3^
3 days	neg *
15–29 days	neg

* No mortality after inoculation of undiluted supernatant.

**Table 4:** Seropositivity among sentinel mice exposed in arthropod-proof cages (APC) and open cages (OC) in Kaeng Khoi cave, 1973–1974.

						Antibody Titer of Positive Mice		
Exposure Room	Type of Cage	Dates of Exposure (Month/Day)	Total Days Exposed	pc/tc ^1^	p/t ^2^ (% pos)	8	40	200
1	OC	6/22–6/26	2.8	7/7	18/32 (56)	18 ^3^		
	OC	3/03–3/28	19	5/5	21/21 (100)	1	2	18
	OC	1/19–2/06	18	3/3	3/11 (27)	3 ^3^		
2	OC	6/22–6/26	2.8	4/4	9/31 (29)			
1	APC	3/03–3/26	19	3/3	8/23 (35)	4	3	1

^1^ Cages with at least one antibody-positive animal/Total cages. ^2^ Number of antibody-positive mice/Total number of mice exposed. ^3^ Sera screened at a 1:8 dilution only.

## Data Availability

All available data are provided in the manuscript and Supplementary Materials.

## Supplementary Materials

The following are available online at https://www.mdpi.com/article/10.3390/microorganisms9102022/s1, Figure S1: Arthropod-proof cages used to house sentinel mice inside Kaeng Khoi cave; Figure S2: Design of the arthropod-proof cage; Figure S3: The field director Mr. Lung (Uncle) Noi and his wife, 1969–1970. Table S1: Animal species associated with and around the entrance to Kaeng Khoi cave; Table S2: Antigens with which immune ascitic fluid to Kaeng Kahoi strain, S-19-B was compared in CF test; Table S3: Grouping ascitic fluids compared with Kaeng Khoi strain, S-19-B, antigen in CF test.

## Appendix A

### Appendix A.1. Characterization of Kaeng Khoi Virus

A single pool of the prototype KKV strain, S-19-B, originally isolated from the brain of a dead free-tailed bat in 1969, was used in all the characterization studies. The pool was prepared from the seventh suckling mouse brain passage of KKV. A sucrose-acetone extracted antigen was prepared from this pool and hyperimmune ascitic fluid against S-19- B was prepared in mice. The CF antibody titer of the ascitic fluid was 512 against S-19-B and the titer of S-19-B antigen was 64.

The 102 viral isolations made from dead bats during the 1969 study were tested by CF for their relationship to KKV. Crude or acetone extracted antigens from infected mouse brains were tested at a 1:8 dilution against a 1:4 dilution of S-19-B immune ascitic fluid and ascitic fluids prepared against 20 of the 102 isolated viruses were tested with the S-19-B extracted antigen. All of the antigens prepared from the 102 isolated viruses reacted with S-19-B immune ascitic fluid in CF tests showing an antigenic relation between S-19-B and the 102 viral isolates. The 20 ascitic fluids prepared all reacted with S-19-B extracted antigen. The results show that all of the 102 isolates were related to or identical to KKV.

### Appendix A.2. Sensitivity to Sodium Deoxycholate (SDC)

Kaeng Khoi virus was sensitive to SDC. Treatment of KKV with SDC reduced the infectivity by more than 2.6 log_10_ SMLD50 (lethal dose for 50% of the suckling mice). The titer of yellow fever virus was similarly reduced, whereas both mouse encephalomyocarditis virus and Theiler’s murine encephalomyelitis virus (strain GD VII), were not decreased in titer after treatment with SDC. Sensitivity to this detergent is expected for bunyaviruses [45], as SDC is known to disrupt lipid bilayers [46].

### Appendix A.3. Estimation of Size of Kaeng Khoi Virions

The virus passed Millipore membranes of 450 and 220 nm. with almost no loss of titer. The virus titer decreased by 1. 0 and 3.6 log_10_ SMLD 50 after filtration through 100 and 50 nm membranes, respectively, indicating that the virion size of KKV was between 50 and 100 nm.

### Appendix A.4. Pathogenicity and Infectivity

The LD 50 (lethal dose for 50% of the animals) of KKV for infant mice inoculated intracerebrally (i.c.) or intraperitoneally (i.p.) and adult mice i.c. was comparable. Only high concentrations of virus killed adult mice by the i.p. route of inoculation. The virus was cytopathic for cell lines: Vero, LLC-MK2 and BKH21 and produced plaques in vero and LLC-MK2 cells. The virus multiplied in a cell line derived from *Aedes albopictus* mosquitoes.

### Appendix A.5. Serological Characterization

No serological cross-reactions were detected when the 1:4 dilution of ascitic fluid of KKV was tested by CF tests against 1:4 dilutions of antigens of viruses of the following groups: arenaviruses, bunyaviruses, herpesviruses, orbiviruses, paramyxoviruses, picornaviruses, poxviruses, reoviruses, rhabdoviruses, togaviruses and to 67 ungrouped viruses and six minor grouped viruses, available at YARU, but unclassified. In addition, KKV antigens did not react to CF tests with ascitic fluids to 24 viral serological groups nor with ascitic fluids to the following viruses: herpes simplex, rabies, LCM, vaccinia, Newcastle disease virus, blue tongue viruses, Bwamba, Pongola, Nyauslo, Eretmapodites, Mossuril, Kamese and four unclassified viruses. The complete virus list for which serological cross-reactivity was evaluated is provide in Tables S2 and S3.

### Appendix A.6. Identification by Thin Section Electron Microscopic Examination

The S-19-B strain of KKV was identified by electron microscopy as a bunyavirus-like particle, enveloped, with a 95–105 nm diameter [1]. Thin section electron microscopy of KKV in mouse brains revealed spherical virions about 100 nm in diameter in the neurons. Virus particle maturation occurred via budding into cisternae of Golgi organelles [47]. The virions were located in the Golgi complex, usually singly or in small groups.

### Appendix A.7. Identification by Structural Polypeptide Analysis

Polyacrylamide gel electrophoresis of degraded virions revealed three polypeptides with approximate molecular weights of 83,000, 30,000, and 20,000.

### Appendix A.8. Virus Isolation Procedures

Vero cell cultures (African green monkey kidney) and/or Swiss mice obtained from SEATO’ s mouse colony were used in virus isolation attempts. Vero cells were propagated in Blake bottles. Monolayers were removed with typsin-versene, resuspended to a final concentration of 10 5 cells/mL in GM. GM contains medium 199 with 1.4 g NaHCO_3_ per liter, and was supplemented with 10% heat-inactivated fetal bovine serum, 100 units penicillin/mL and 10 μg streptomycin/mL. Tissue culture tubes and l oz. prescription bottles were seeded with 0.9 mL and 4 mL of cell suspensions, respectively, and incubated for four days at 37 °C prior to inoculation.

Individual tissues were triturated in sterile, 7 mL Ten Broeck tissue grinders containing 2 mL GM. The resultant suspension was centrifuged at 9750× *g* for 30 min at 4 °C. The supernatant was withdrawn and divided into two parts; one portion was frozen at −60 °C for re-isolation attempts, and the other one was inoculated intracerebrally (i.c.) into a litter of newborn Swiss mice (0.02 mL per mouse) and/or into 3 tubes of Vero cells (0.1 mL/tube). Urine, oral swabs and tissues from morbid bats were inoculated into Vero cell cultures only. Drained cell culture tubes were inoculated with 0.l mL of supernatant and incubated for 1 h at 37 °C. After adsorption, maintenance medium (l mL) was added. Maintenance medium (MM) was GM supplemented with 5% fetal bovine serum. The inoculated cultures were incubated at 37 °C, and were examined periodically for virus cytopathic effect (CPE) for a period of 14 days. Supernatants from tubes showing CPE were subpassaged. Maintenance medium was changed every third day. Inoculated mice were observed for 15 days. Brains of those found sick or dead were harvested and subpassaged by intracerebral inoculation of mice. Virus isolates were identified by neutralization (N) tests, using Kaeng Khoi hyperimmune rabbit serum or mouse ascitic fluid. These tests were done with constant serum and varying virus dilutions.
