# Supplementary material for: Investigations on Vector-Borne and Aerosol Transmission Potential of Kaeng Khoi Virus in Cave-Dwelling Wrinkle-Lipped Free-Tailed Bats (Chaerephon plicatus) in Thailand
Source: Microorganisms. 2021 Sep 24;9(10):2022. doi: 10.3390/microorganisms9102022 (PMC8538812; doi:10.3390/microorganisms9102022)
Supplement: Supplementary file 1 [file microorganisms-09-02022-s001.zip › microorganisms-1366805-supplementary.pdf]

Table S1. Animal species associated with and around the entrance to Kaeng Khoi cave.

**Species Inside the Cave**	
Arthropods	*Associated Mammal Host*
Flea (*Lagaropsylla macula*)	Wrinkle-lipped free-tailed bat (*Chaerephon plicatus*; *Tadarida plicata plicata*)
Oriental rat flea (*Xenopsylla cheopis*)	Roof rat (*Rattus rattus*)
Tick (*Ixodes kopsteini*)	Wrinkle-lipped free-tailed bat (*Chaerephon plicatus*; *Tadarida plicata plicata*)
Bat flies, family Streblidae (*Brachytarsina macrops*)	Tomb bat (*Taphozous theobaldi*)
Bugs, family Cimicidae (*Cimex insuetus*; *Stricticimex parvus*)	*C. plicatus, T. theobaldi*
**Species Outside the Cave**	
Neill's giant dwarf rat (*Leopoldamys neilli*)	
Limestone rat (*Niviventer hinpoon*)	
Roof rat (*Rattus rattus*)	
Shikra (*Accipiter badius*) – preys on bats	
Oriental hobby (*Falco severus*) – preys on bats	
Brown hawk owl (*Ninox scutulata*) – preys on bats	

Figure S1. Arthropod-proof cages used to house sentinel mice inside Kaeng Khoi cave.

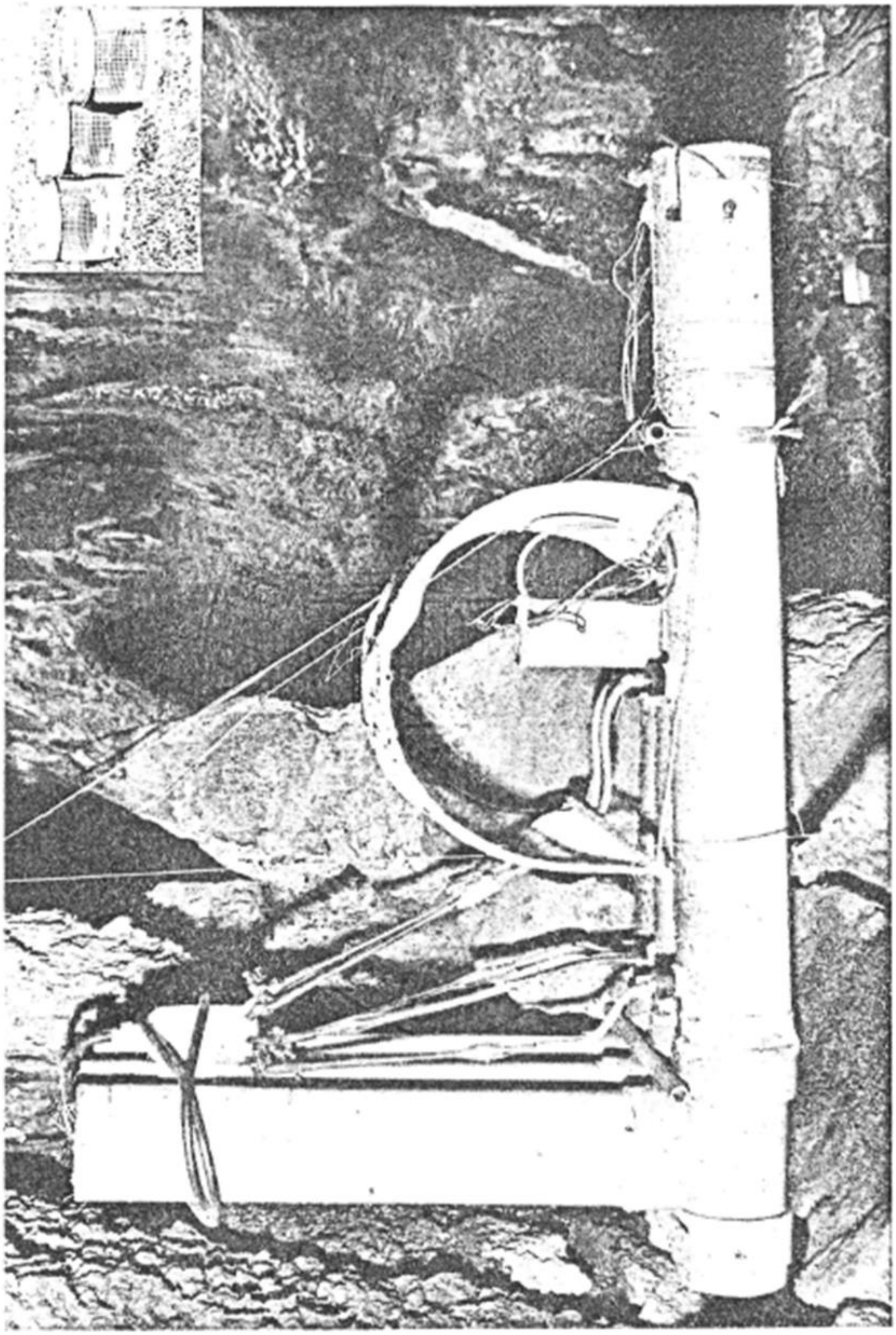

Figure S2. Design of the Arthropod-proof cage.

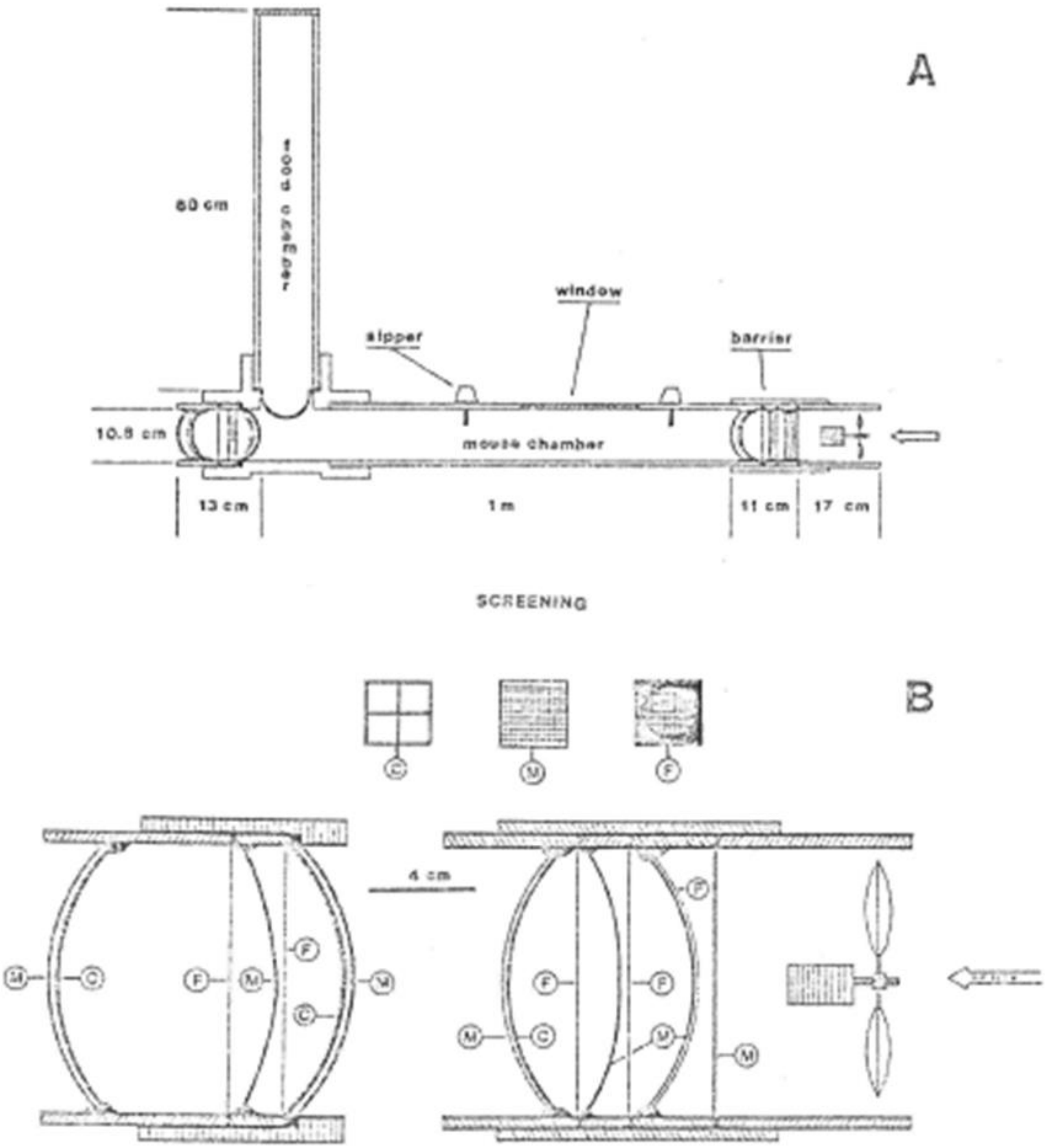

Figure S3. The field director Mr. Lung (Uncle) Noi and his wife, 1969 – 1970.

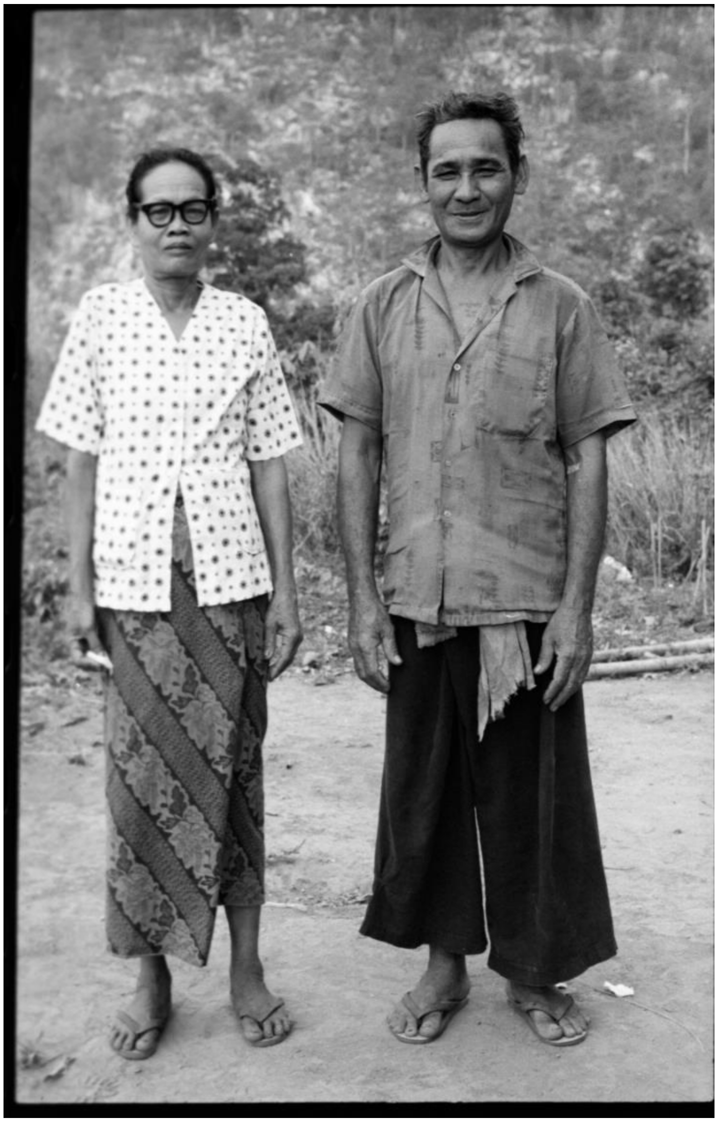

Table S2. Antigens with which immune ascitic fluid to Kaeng Kahoi strain, S-19-B was compared in CF test

TABLE AI. Antigens with which immune ascitic fluid to Kaeng Khoi strain, S-19-B, was compared in CF test[1]

ARENAVIRUSES

Tacaribe
Junin, LCM, Pichinde, Tacaribe, Tamiami

BUNYAVIRIDAE

BUNYAVIRUS

Bunyamwera
Ilesha, Kairi, Sororoca, Tensaw, Wyeomyia

Bwamba
Bwamba, Pongola

C Serogroup
Apeu, Caraparu, Nepuyo, Oriboca

Capim
Acara, BeAn 84381, Bushbush, Capim, Guajara

California
Melao, Tahyna, Trivittatus

Guama
Bertioga, Guama

Koongol
Wongol

Patois
Patois

Simbu
Buttonwillow, Manzanilla, Simbu, Sathuperi, Utinga

Tete
Bahig, Tete

Olifantsvlei
Olifantsvlei

Unassigned
Gamboa, Mirim, BeAn 141106

[1]Antigens were provided by the Yale Arbovirus Research Unit (YARU)

TABLE AI (continued)

BUNYAVIRIDAE (continued)

SEROLOGICALLY UNRELATED BUNYAVIRIDAE VIRUSES

Anopheles A
    Anopheles A, Lukuni, Tacaiuma

Anopheles B
    Anopheles B, Boracea

Bakau
    Bakau

Bhanja
    Bhanja

Congo
    Congo, Hazara

Ganjam
    Dugbe, Ganjam

Kaisodi
    Kaisodi, Lanjan, Silverwater

Lone Star
    Lone Star

Mapputta
    Mapputta, Trubaman

Pacui
    Pacui

Phlebotomus Fever
    Arumowot, Anhanga, BeAn 100049, Bujaru, Candiru, Chagres, Icoaraci, Itaporanga, Punta Toro

Tataguine
    Tataguine

Thogoto
    Thogoto

Turlock
    Turlock, Umbre, Yaba 1

TABLE AI (continued)

BUNYAVIRIDAE (continued)

Uukuniemi
EgAn 1825-61, Grand Arbaud, Uukuniemi

Witwatersrand
Witwatersrand

HERPESVIRUS

Herpes simplex

ORBIVIRUSES

Eubenangee
Acado, Eubenangee, Pata, Epizootic haemorrhagic disease of deer, N.J. strain IbAr 22619

Corriparta
Corriparta

Changuinola
Changuinola, Irituia

Kemerovo
Chenuda, Kemerovo, Tribec, Wad Medani, Mono Lake

Palyan
Palyan

Lebombo
Lebombo

Colorado Tick Fever
Colorado Tick Fever

Blue Tongue
Blue Tongue

PARAMYXOVIRUSES

Newcastle disease
Nariva

TABLE AI (continued)

PICORNAVIRIDAE

Encephalomyocarditis (EMC-Kissling)
Mouse (GD-7) Polio

POXVIRUSES

Cotia
Ectromelia

REOVIRUS

Reovirus Type 3

RHABDOVIRUSES

Vesicular Stomatitis
Piry, VS-Indiana, VA-New Jersey

Rabies
Lagos Bat, IbAn 27377, Rabies

Kern Canyon
Kern Canyon

Mt. Elgon Bat
Mt. Elgon Bat

Hart Park
Hart Park

TOGAVIRIDAE

Alphavirus
EEE, WEE, Sindbus, Chikungunya

Flavivirus
SLE, Powassan, Montano Myotis Leukoencephalitis (MML), Yellow Fever, Louping Ill, West Nile, Zika, Kadam, Ntaya

UNCLASSIFIED VIRUSES

MINOR SEROLOGICAL GROUPS

Nyando
Eretmapodites 147, Nyando

TABLE AI (continued)

UNCLASSIFIED VIRUSES (continued)

Mossuril
Kamese, Mossuril

Hughes
Farallon, Hughes, Soldada Rock

Quaranfil
Johnston Atoll, Quaranfil, Napal Tick

Qalyub
Bandia, Qalyub

Timbo
Chaco, Timbo

SEROLOGICALLY UNGROUPED VIRUSES

Aruac
Aus MK 7532
Aus CH 9824
Aus CH 9935
Aus MRM 10434
Aus MK 6357
Aus MK 7937

BeAn 67949
Be Ar 106435
Boteke
Botembe

Burg el Arab

Ch 9935
Conn An 114

DGK

Eg An 1398-61
Eg Art 904
Eg Art 1237
Eg Art 1113

Germiston
Gossas

I 6235
I 66416
I 61-2629
I 66415
I 58
I 81
I 66413
I 66413
Ib An 10065
Ib An 28946
Ib An 17854
Ib An 17143
Ib Ar 23380
Ib H 11306
Ieri

J 19
J 134
Jurona

Klamath
Kowanyama

TABLE AI (continued)

UNCLASSIFIED VIRUSES (continued)

SEROLOGICALLY UNGROUPED VIRUSES (continued)

M 22 68a
Marco
Matariya
Minatitlan (Mex 67 U5)
Minnal
MM 314
MP 359
MRM 40434
MRM 4059

Naples
Navarro (Calif. 874)
Nyamanini

Okola
Oyo

Pak Argas 461

Q 3255
Sa An 3518
Sawgrass
Sicilian
Sli 763
Sud Ar 1169-64

Tembe
Thimiri
Triniti

Upolu

YM 31

Table S3. Grouping ascitic fluids compared with Kaeng Khoi strain, S-19-B, antigen in CF test

TABLE AII. Grouping ascitic fluids compared with Kaeng Khoi strain, S-19-B, antigen in CF test[1]

POLYVALENT ASCITIC FLUIDS
Group A
Group B
Group C
Group Bunyamwera
Group Simbu
Group Capim
Polyvalent Anopheles A etc. G211-601-567
Group Bwamba
Poly. Herpes, Rabies, LCM, Vaccinia, NDV
Group California
Group Guama
Polyvalent Quarenfil-Kaisodi-Qalyub
Group Tacaribe
Group VSV
Group Patois
Group Phlebotomus
Group Kemerovo
Polyvalent Palyam
Group Tete
Group Hart Park
Group Nyamanini
Group Tembe
Group Kwatta
Group Triniti
Group Jurona
Blue Tongue

[1]Grouping ascitic fluids were provided by the Yale Arbovirus Research Unit (YARU)
